# Identification of ncRNAs as potential therapeutic targets in multiple sclerosis through differential ncRNA – mRNA network analysis

**DOI:** 10.1186/s12864-015-1396-5

**Published:** 2015-03-28

**Authors:** Haritz Irizar, Maider Muñoz-Culla, Matías Sáenz-Cuesta, Iñaki Osorio-Querejeta, Lucía Sepúlveda, Tamara Castillo-Triviño, Alvaro Prada, Adolfo Lopez de Munain, Javier Olascoaga, David Otaegui

**Affiliations:** Multiple Sclerosis group, Biodonostia Health Research Institute, Paseo Dr. Begiristain s/n, San Sebastián, 20001 Spain; Spanish Network on Multiple Sclerosis (REEM), San Sebastián, Spain; Spanish Network on Multiple Sclerosis (REEM) and Immunology Department, Donostia University Hospital, San Sebastián, Spain; Spanish Network on Multiple Sclerosis (REEM) and Neurology Department, Donostia University Hospital, San Sebastián, Spain; Immunology Department, Donostia University Hospital, San Sebastián, Spain; Biodonostia Health Research Institute, San Sebastián, Spain; Department of Neurology, Donostia University Hospital, Donostia – San Sebastián, Spain; Centro de Investigación Biomédica en red Enfermedades Neurodegenerativas (CIBERNED) and Department of Neuroscience, University of the Basque Country (UVP/EHU), San Sebastián, Spain

**Keywords:** Multiple sclerosis, ncRNA, snoRNA, miRNA, Coexpression, Differential network, Therapeutic target, Rewiring, ACA40

## Abstract

**Background:**

Several studies have revealed a potential role for both small nucleolar RNAs (snoRNAs) and microRNAs (miRNAs) in the physiopathology of relapsing-remitting multiple sclerosis (RRMS). This potential implication has been mainly described through differential expression studies. However, it has been suggested that, in order to extract additional information from large-scale expression experiments, differential expression studies must be complemented with differential network studies. Thus, the present work is aimed at the identification of potential therapeutic ncRNA targets for RRMS through differential network analysis of ncRNA – mRNA coexpression networks. ncRNA – mRNA coexpression networks have been constructed from both selected ncRNA (specifically miRNAs, snoRNAs and sdRNAs) and mRNA large-scale expression data obtained from 22 patients in relapse, the same 22 patients in remission and 22 healthy controls. Condition-specific (relapse, remission and healthy) networks have been built and compared to identify the parts of the system most affected by perturbation and aid the identification of potential therapeutic targets among the ncRNAs.

**Results:**

All the coexpression networks we built present a scale-free topology and many snoRNAs are shown to have a prominent role in their architecture. The differential network analysis (relapse vs. remission vs. controls’ networks) has revealed that, although both network topology and the majority of the genes are maintained, few ncRNA – mRNA links appear in more than one network. We have selected as potential therapeutic targets the ncRNAs that appear in the disease-specific network and were found to be differentially expressed in a previous study.

**Conclusions:**

Our results suggest that the diseased state of RRMS has a strong impact on the ncRNA – mRNA network of peripheral blood leukocytes, as a massive rewiring of the network happens between conditions. Our findings also indicate that the role snoRNAs have in targeted gene silencing is a widespread phenomenon. Finally, among the potential therapeutic target ncRNAs, SNORA40 seems to be the most promising candidate.

**Electronic supplementary material:**

The online version of this article (doi:10.1186/s12864-015-1396-5) contains supplementary material, which is available to authorized users.

## Background

The huge development of molecular biology in the last few decades has allowed us to observe how, as we have gained insight into the biology of the cell, the regulation of gene expression has revealed more and more complexity around the central **DNA → RNA → protein** axis that provides the backbone to our view of molecular biology. Additional levels of regulation and new players have appeared with substantial roles on the regulation of the expression of our genes. During the last decade, it has become evident that small non-coding RNAs (sncRNAs) participate in widespread and essential regulatory mechanisms in most eukaryotic cells. Novel classes of small RNAs, their biogenesis pathways and cellular effects are continuously being described, and new properties of already established sncRNAs are still being discovered [1]. A broad range of sncRNA types have already been characterized (microRNAs, siRNAs, piRNAs etc.) [1,2] but, among them, the microRNAs (miRNAs) have occupied an indisputable central position since their characterization as a distinct class of biological regulators with conserved functions in the early 2000s [3-5].

MicroRNAs regulate gene expression at the post-transcriptional level and have been shown to be involved in almost all biological processes like development, cell differentiation, proliferation and cell death and also in several pathological events like cancer, neurodegeneration or autoimmunity [6-9]. Among other autoimmune diseases, alterations in miRNAs have been related to multiple sclerosis (MS), as in the last years several works have studied miRNA expression in a variety of tissues (peripheral blood, brain and CSF) from MS patients and in the Experimental Autoimmune Encephalitis (EAE) animal model (reviewed in [10]). All these studies found alterations in miRNA expression levels in MS patients compared to healthy controls and therefore, an implication for miRNAs seems to be evident. Yet, little overlap is observed among different studies, probably due to the difference in the miRNA profiling technology, the complexity of the tissue being studied and the relatively small sample size of all studies.

In addition to the miRNAs, the small nucleolar RNAs (snoRNAs) have also been shown to have important functions in cell homeostasis. snoRNAs are 60 to 300 nucleotide-long ncRNAs that are generally required for alternative splicing and RNA modifications. Two main types of snoRNAs have been described, the box C/D snoRNAs (SNORDs), implicated in the guidance of 2’o-ribose-methylation of ribosomal RNA (rRNA), and the box H/ACA snoRNAs (SNORAs) that guide pseudouridylation of target rRNAs. Although it was thought that snoRNAs were involved only in alternative splicing and rRNA modification, a good amount of evidence indicates that they can exert their gene expression regulatory effect by specifically targeting other RNAs, such as small-nuclear RNAs (snRNAs) and mRNAs, thanks to their capacity to function as precursors of the so called snoRNA-derived RNAs (sdRNAs) [11]. As for microRNA, snoRNA has been also identified as differentially expressed in some diseases, including MS [12,13].

The vast majority of the studies aimed at the identification of relevant miRNAs for a disease or a condition using miRNA expression data, have done so by analyzing differential expression. However, the involvement of sncRNAs in a disease or a specific condition can be studied not only through the analysis of differential expression, but also through the characterization of the alterations of the sncRNA – mRNA regulatory network. The description of the alterations this network presents in association to a certain phenotype can lead to the identification of the most relevant sncRNAs driving these alterations and provide additional information to determine the sncRNAs that could have a central role in the disease.

The construction of miRNA – mRNA networks has already been used for several purposes such as shedding light on the role of miRNAs in cancer [14-16], studying the effect of infections on host miRNA – mRNA networks [17,18] or analyzing the rewiring of the transcriptional regulation network in the allogeneic response of T cells [19]. However, in most of these works, miRNA – mRNA networks (built from expression data for both miRNAs and mRNAs in some works, using computational miRNA target-prediction algorithms in others and applying both approaches in some few cases) were used to provide a regulatory context to differential expression analysis results by either building the networks only for the differentially expressed miRNAs/mRNAs or by mapping the fold-changes obtained for those transcripts in a global network. From the works referenced above, only one [16] used differential network analysis to describe the effect of cancer in miRNA – mRNA networks, showing that, although the structure of the network was not altered, the strength of the connections changed in cancer patients when compared to healthy controls.

In the present work, we have used a differential network analysis approach to, on one hand, understand the alterations that the diseased status of MS produces in the ncRNA – mRNA coexpression network and, on the other hand, identify the most relevant ncRNAs for MS physiopathology. ncRNA – mRNA coexpression networks have been constructed from both ncRNA (specifically miRNAs, snoRNAs and sdRNAs) and mRNA large-scale expression data obtained from patients in relapse, the same patients in remission and healthy controls. Condition-specific (relapse, remission and healthy) networks have been built and compared to identify the parts of the system most affected by perturbation and aid the identification of potential therapeutic targets among the selected ncRNAs.

## Methods

### Design of the study

**Figure 1:**
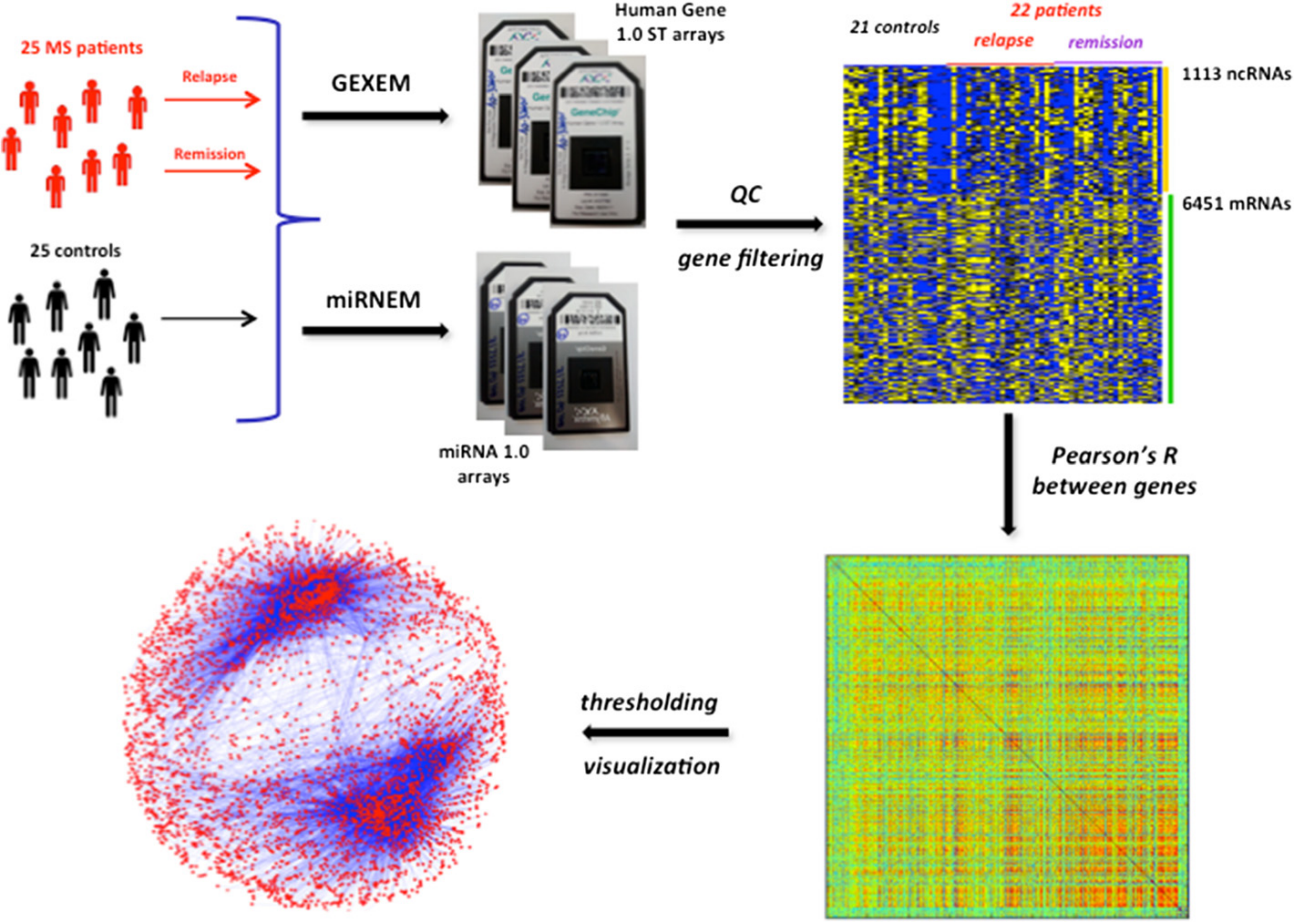
**Design of the study.** The gene expression data come from two studies performed in parallel in the laboratory: GEXEM and miRNEM. Large scale mRNA expression and miRNA expression have been measured on RNA isolated from peripheral blood leukocytes using, respectively, the Human Gene 1.0 ST and the miRNA 1.0 arrays by Affymetrix. The removal of some samples for quality control issues and the filtering of genes have yielded a matrix with gene expression data from 65 samples and 7564 genes (1113 ncRNAs and 6451 mRNAs). Pearson’s R has been computed between all pairs of genes and, after thresholding (through the elimination all mRNA – mRNA and ncRNA – ncRNA correlations and the correlations below a threshold │R│), the resulting network has been visualized in Cytoscape.

The gene expression data for the present study was collected from two studies performed in parallel in our laboratory (Figure 1). In one of them (from now on the GEXEM study) the whole genome mRNA expression analysis of 22 MS patients in remission and relapse and 22 age and sex-matched healthy controls was performed [20]. In the other study (from now on the miRNEM study) the expression of selected ncRNAs was analyzed in the same samples. In total, after the quality control, 65 samples, obtained from 22 patients in relapse and remission and 21 healthy controls (Additional file 1: Table S1), had both mRNA and ncRNA expression data. The mRNA and ncRNA expression data from those 65 samples and the results of the differential expression analysis of the miRNEM study were used in the present work.

### Subjects and sample collection

All MS patients were diagnosed with Relapsing-Remitting Multiple Sclerosis according to McDonald criteria [21]. Two peripheral blood samples were collected from each MS patient, one sample in relapse and one in remission; only one sample was collected for each control subject. Relapse was defined as the development of new or recurrent neurological symptoms not associated with fever or infection lasting for at least 24 hours and accompanied by new neurological sign, following a period of symptomatic stability of 30 days [22]. Peripheral blood of patients and healthy controls was obtained at the Neurology Department of Donostia University Hospital. Blood extraction was performed in the early morning for controls and remission samples and at arrival of the patient for relapse samples. In this last case, blood extraction always preceded the administration of steroids for relapses. RNA extraction was carried out no more than 2 hours after the blood was collected and during this time blood samples were kept at 4°C. In all the cases, 10 ml of blood were collected in EDTA tubes by venipuncture. All the procedures have been approved by the hospital’s ethic committee (*Comité ético de investigación Clínica del area sanitaria de Gipuzkoa*/Ethic committee of Clinical research in the Health area of Gipuzkoa). Written informed consent was received from participants prior their inclusion in the study, where they authorized the publication of their age, sex and clinical details in scientific articles and meetings.

### RNA isolation

RNA extraction from leukocytes was performed using the LeucoLOCK™ Total RNA Isolation System by Ambion with the alternative protocol to recover Total RNA. RNA samples were aliquoted and stored at −80°C for later use. Samples were extracted and stored at the Donostia – San Sebastián node of the Basque Biobank (www.biobancovasco.com). Before the gene expression analysis, RNA integrity was checked with the Agilent RNA 6000 Nano kit and the samples with an RNA Integrity value (RIN) above 6 were accepted to be further processed.

### The GEXEM study

Whole genome gene expression of the samples was measured by the Human Gene 1.0 ST Affymetrix microarray. 300 ng of total RNA were used for microarray analysis following the manufacturer’s instructions. Both the WT Expression Kit by Ambion and the GeneChip Hybridization Wash and Stain kit by Affymetrix were used in the process. Briefly, during the three-day protocol, complementary single-strand DNA was synthesized from RNA, to be later fragmented, labeled and hybridized during 16 hours. The hybridized microarrays were washed and stained in a GeneChip Fluidics Station 450 and scanned in a GeneChip 7G Scanner afterwards.

The data of the .cel files were normalized with the Robust Multichip Average (RMA) in the Expression Console software by Affymetrix. As multiple-testing correction makes the group-wise comparisons more stringent as more genes are included in the analysis, we filtered the data to remove the least informative genes using the BRB-Array Tools software [BRB-ArrayTools Development Team, version 4.2.1] implemented in Microsoft Excel [Microsoft Corporation, Microsoft Office Professional Edition 2003]. The genes with a 90^th^ percentile value smaller than 55.7 (the mean intensity of the negative control probes) were removed and 6451 probesets were left for the subsequent analysis.

### The miRNEM study

The selected ncRNA expression analysis was done with the GeneChip miRNA array v1 by Affymetrix. 500 ng of total RNA were used for microarray analysis following the manufacturer’s instructions. The quality control, summarization and normalization of ncRNA expression data was done with the miRNA QC Tool v.1.0.33.0 software by Affymetrix. Whether the detection of the expression for each probe was significant or not was determined by comparing them against the anti-genomic probes present in the array using a Wilcoxon test for the miRNA probesets and the Affymetrix Power Tool for the rest (snoRNA and sdRNA probesets). Afterwards, background correction and quantile-based normalization of the data were done. The summarization of the data was performed with the RMA algorithm. Finally, in order to reduce the dimensionality of the dataset, the non-human probes present in the array and the probes not detected in any of the samples were filtered out. After these two filtering steps, 1113 probes were left for the subsequent analysis.

The differential expression analysis was performed with the MEV 4.7.2 software [23,24]. The dataset was subdivided based on treatment and two comparisons were done for each subgroup: relapse vs. remission and remission vs. controls. For the identification of the differentially expressed ncRNAs, the Rank Products algorithm was used [25]. This procedure resulted in a 25 probe-list with ncRNAs altered in relapse (vs. remission) and a 53 probe-list with ncRNAs altered in remission (vs. controls).

### Coexpression network analysis

Before the construction of gene expression correlation matrices, and in order to make the data obtained from each study comparable, a standardization of the data was done. For that, z-scores were calculated for all gene expression values (including mRNA and ncRNA data) and those were used for the subsequent coexpression analysis. Coexpression matrices were created in Pylab by computing pairwise Pearson’s R between all the gene-pairs. From a gene expression dataset with *x* genes and *y* samples, *x*^2^ pairwise Pearson’s R correlations were computed using, in each case, two expression vectors with length *y*. In each of the different coexpression analyses, the threshold to cut the matrix was established considering the length of the vectors used in the computation of R, i.e. the number of expression values for each gene (*y*). The resulting matrix was transformed into a column-format network with the following structure: gene 1 – correlation value – gene 2. The visualization and analysis of the resulting networks was performed in Cytoscape 3.0.1 [26].

## Results

### Global network

**Figure 2:**
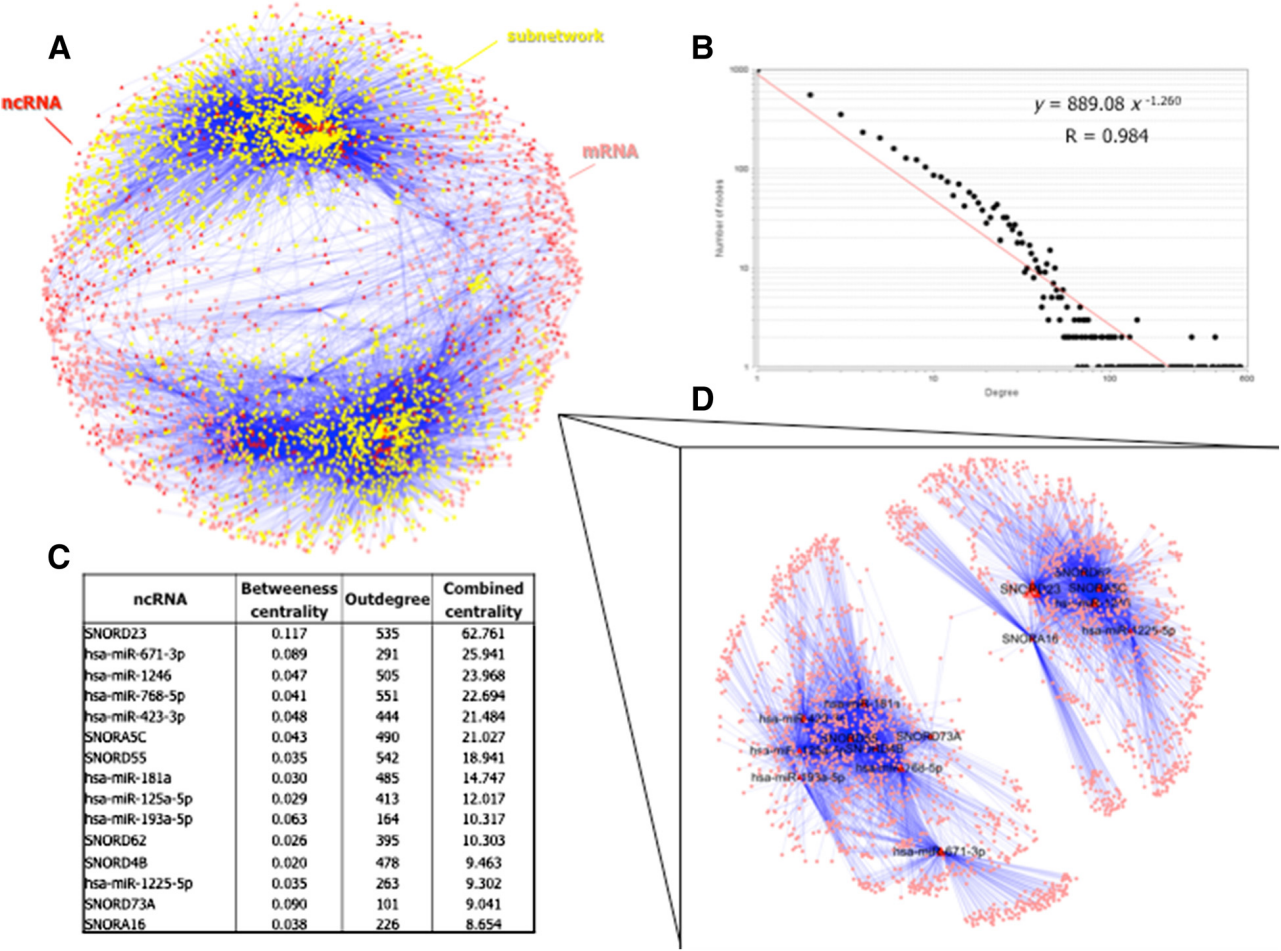
**Global ncRNA – mRNA coexpression network.** The global network presents an only fully-connected component **(A)** wired by ncRNA – mRNA correlations below −0.42. The node degree distribution is represented in a logarithmic scale in both axes and fits a negative power law **(B)**. The 15 nodes (ncRNAs all of them) with a combined centrality value (betweeness centrality * outdegree) above 8.6 (percentile 0.975) **(C)** and their first neighbors form the core subnetwork **(D)**.

In order to gain insight into the general structure of the ncRNA – mRNA network, a global coexpression network has been inferred from the gene expression data from the 65 samples and the 7564 transcripts (6451 mRNAs and 1113 ncRNAs). Under the assumption that miRNAs downregulate mRNA expression by either blocking translation or promoting degradation and considering the evidence that snoRNAs, through their role as sdRNA precursors, might exert a similar effect on mRNA expression, only the negative correlation values between the selected ncRNAs and mRNAs were selected. Thus, taking into account the length of the vectors used for computing correlations (i.e. number of samples (N) = 65), the matrix obtained in the correlation analysis was cut at Pearson’s R < −0.42 (p = 0.0005) and all the mRNA – mRNA and ncRNA – ncRNA correlations (the *intra-array correlations*) were removed. The resulting network (wired by negative ncRNA – mRNA correlations only) presents one fully connected component composed of 4101 nodes (3489 mRNAs (54.08% of all mRNAs) and 612 ncRNAs (54.99% of all ncRNAs)) and 29600 negative correlations (ranging from −0.42 to −0.7) (Figure 2A). In contrast, the coexpression network wired by positive ncRNA – mRNA correlations (Pearson’s R > 0.42) is composed by 6069 nodes and 56461 edges (Additional file 1: Figure S1).

The node-degree distribution of the global coexpression network fits a negative power law (y = 889.08 x^-1.26^; where *y* is the number of nodes and *x* is the degree) indicating a scale-free topology (Figure 2B). Besides, a combined centrality measure has been computed for all nodes by multiplying the outdegree (the number of edges that originate from a node) and the betweeness centrality (the fraction of all shortest paths of the network that pass through that particular node). This combined centrality has been computed with the aim of obtaining a parameter that reflects both the strength of the effect of each ncRNA (how many genes it targets) and its importance on the global connectivity of the network. The nodes with a combined centrality above 8.6 (97.5^th^ percentile) are 15 ncRNAs (6 snoRNAs and 9 miRNAs) (Figure 2C) and, along with their first neighbors (2176 genes), form the core of the global network (Figure 2D).

### Status-specific networks

With the aim of testing the effect that disease status (patients in relapse, patients in remission or healthy controls) exerts on the global ncRNA – mRNA coexpression pattern, status-specific networks were created. As for the global network, only negative ncRNA – mRNA correlations were included but, in this case, accordingly to the lower length of the vectors used in the computation (i.e. smaller sample size; 22 for relapse and remission and 21 for controls), more stringent Pearson’s R cutoff thresholds were used (R < − 0.537 for relapse and remission; R < − 0.549 for controls; p = 0.01 in all cases).

**Figure 3:**
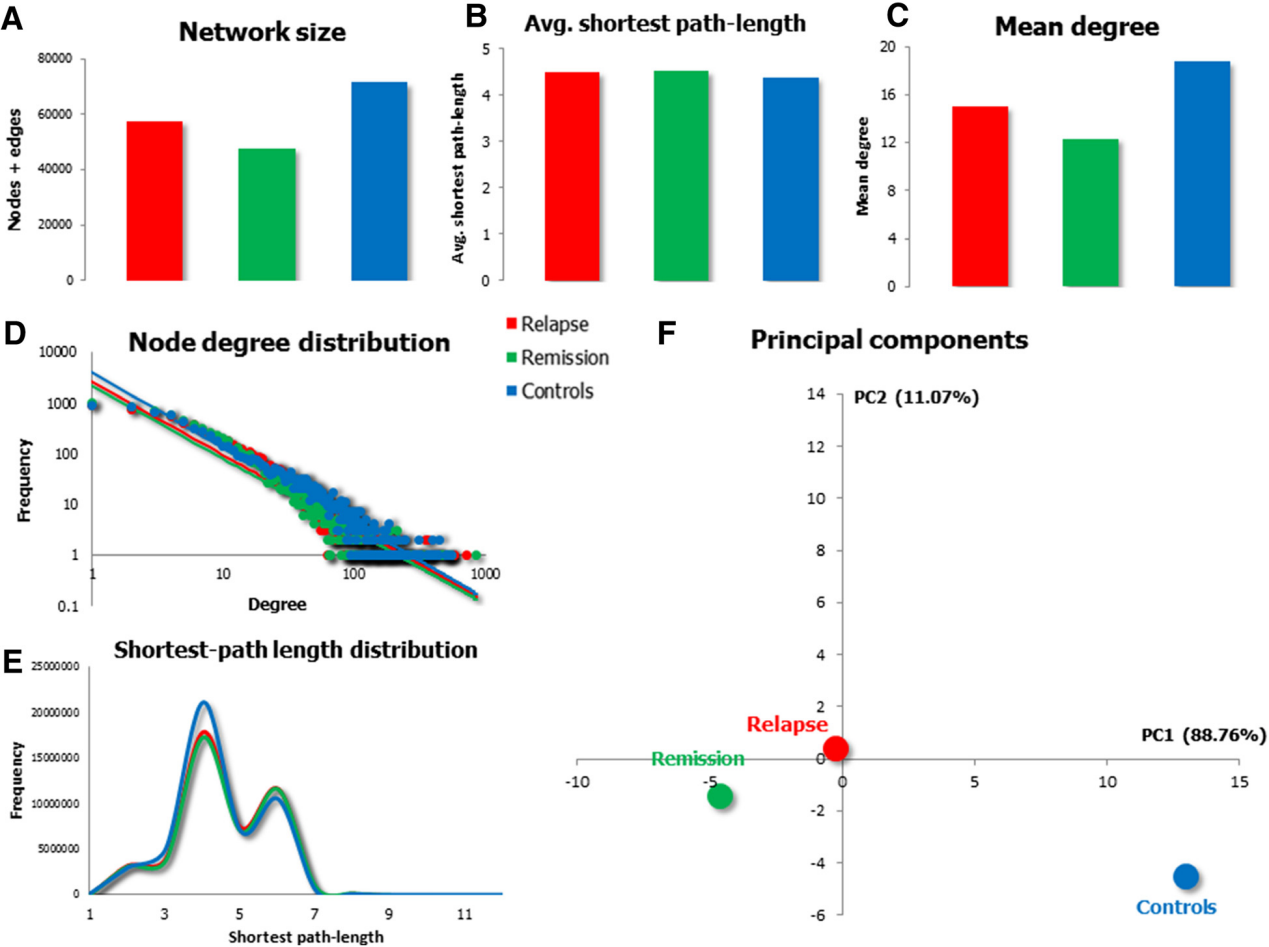
**Status-specific ncRNA-mRNA network comparison based on parameters describing network topology.** The size of the network **(A)**, the average shortest-path length **(B)** and the average degree **(C)** have been calculated for each network. The node degree distribution of the three networks follows a negative power law **(D)**, indicating a scale-free topology and the arguments are very similar in the three cases (−1.439 for relapse, −1.431 for remission and −1.484 for controls). The frequency distributions of the shortest path-length of the three networks have also been plotted **(E)**. Finally, the similarity of the three networks has been estimated with a Principal Component Analysis (PCA). The first two components accounting, respectively, for 88.76% and 11.07% of the variability have been plotted **(F)**. For the PCA, the values of the following network descriptor parameters have been used: number of connected components, network centralization, characteristic path-length, average degree, network heterogeneity and the argument of the power law fitting the node degree distribution.

To check whether the disease status has any influence on the topological characteristics of the ncRNA – mRNA network, several parameters describing network-topology have been calculated and plotted (Figure 3). Three basic descriptors, the total size of the network (number of nodes + edges, Figure 3A), the average shortest path-length (Figure 3B) and the mean degree (Figure 3C), suggest that, regarding topology, the networks inferred from relapse and remission samples present a higher similarity than that obtained from control samples. In general, the controls’ network is larger (includes more nodes and edges) and shows a remarkably higher connectivity (higher mean degree). Nevertheless, despite presenting a higher connectivity, the efficiency for transmission of information (there is transmission of information through an edge that links A and B, if the behavior of variable A (the expression of a miRNA) influences the behavior of variable B (the expression of an mRNA) and/or *vice versa*) is not higher in the controls’ network as the three graphs show a very similar average shortest path-length.

On the other hand, as that of the global network, the frequency distribution of the three status-specific networks fits a negative power law (Figure 3D), indicating a scale-free topology. The correlation values of the power law are not as high as that of the global network (0.821 for relapse, 0.842 for remission and 0.813 for controls) but remain statistically significant (p < 10^−8^ in all cases). The argument is similar for the three distributions (−1.439 for relapse, −1.431 for remission and −1.484 for controls), which suggests that the proportion of hubs and poorly connected nodes is similar in the three networks. The distribution of the shortest path-length between all pairs of nodes (Figure 3E) is also very similar for the three graphs. However, for the two parameters, the relapse and remission distributions seem to be more similar and the one obtained for the controls’ network appears to be the outlier.

In order to get a more accurate idea of the similarity/dissimilarity of the three networks, a Principal Component Analysis (PCA) has been performed using the values obtained for several topology descriptors: number of connected components, network centralization, average path-length, mean degree, network heterogeneity and the argument of the power law. The networks have been plotted based on their values for the two principal components of the PCA analysis, which, respectively, account for 88.76% and 11.07% of the variability (Figure 3F). The results of the PCA indicate that, as suggested by the simple topology descriptors and the distributions of the node-degree and the shortest path-length, the controls’ network is the outlier and, from the point of view of similarity/dissimilarity of network topology, the relapse and remission networks are closer to each other; an unsurprising result considering that the gene expression data for the relapse and remission networks come from the very same patients, while the data for the controls’ network comes from a different population.

Nevertheless, the node degree and shortest path-length distributions of the three networks seem to be very similar, a notion that is supported by the fact that the correlations between the node-degree distributions lie above 0.9998 (p < 10^−8^ in all cases) and those between the shortest path-length distributions range from 0.9881 to 0.9997 (p < 10^−8^ in all cases).

Thus, we hypothesized that, if the topology of the three status-specific networks has similar features, the lists of the top ncRNAs/mRNAs playing a central role in each of the three networks should also overlap. However, from the 65 unique ncRNAs with a combined centrality in the top 2.5% (97.5^th^ percentile), only six (hsa-miR-181a, hsa-miR-423-3p, hsa-miR-1225-5p, hsa-miR-1268, SNORA40 and SNORD23) appear in two lists (Table 1). The rest of the ncRNAs (59) play a central role in only one of the networks. This fact suggests that despite the high similarity of the architecture of the status-specific networks, the specific genes playing central roles are different and, thus, different members of the transcriptome are involved in the core ncRNA – mRNA network in different conditions.

**Table 1:** **Lists of top ncRNAs for each status-specific network**

**Relapse**		**Remission**		**Controls**	
**ncRNA**	**Combined centrality**	**ncRNA**	**Combined centrality**	**ncRNA**	**Combined centrality**
hsa-miR-671-3p	53.70	**hsa-miR-181a**	72.64	hsa-miR-1246	17.56
hsa-miR-744	26.81	SNORA16	25.17	**hsa-miR-181a**	16.42
hsa-miR-99b*	15.75	hsa-miR-768-5p	20.27	SNORD116-8	11.50
**SNORD23**	10.92	SNORD16	17.49	SNORA42	7.19
SNORA58	10.76	hsa-miR-574-5p	13.93	hsa-miR-339-5p	7.08
hsa-miR-331-5p	10.25	SNORD57	13.03	SNORD1C	5.52
SNORA41	7.91	SNORD55	10.25	SNORD4B	4.72
hsa-miR-425	7.45	SNORD42A	8.01	SNORD60	4.55
hsa-miR-1224-5p	6.89	SNORD68	7.56	SNORD80	4.33
hsa-miR-1228	6.79	**hsa-miR-423-3p**	7.41	hsa-miR-671-5p	4.26
**hsa-miR-1225-5p**	5.07	**hsa-miR-1225-5p**	5.29	hsa-miR-708	4.09
**hsa-miR-423-3p**	4.99	hsa-miR-130b	5.29	hsa-miR-484	4.03
SNORA31	4.98	hsa-miR-532-5p	4.81	SNORD18C	4.02
SNORA51	4.91	SNORA6	4.81	SNORD28	4.00
SNORD9	3.84	hsa-miR-194*	4.74	SNORD87	3.84
SNORD115-8	3.75	SNORD116-2	4.51	hsa-miR-342-3p	3.75
SNORD118	3.74	hsa-miR-154*	4.27	hsa-miR-593	3.14
**hsa-miR-1268**	3.48	hsa-miR-1306	4.05	**SNORA40**	3.07
hsa-miR-30b	3.34	SNORD116	3.56	SNORD13	2.81
SNORA5C	3.03	hsa-miR-185	3.53	SNORD29	2.80
**SNORA40**	2.74	hsa-miR-195*	3.48	SNORD44	2.75
SNORA2	2.73	**hsa-miR-1268**	2.73	SCARNA23	2.68
hsa-miR-491-5p	2.70	hsa-miR-346	2.62	hsa-miR-191	2.59
				hsa-miR-15b	2.50
				**SNORD23**	2.43

The top 2.5% (97.5^th^ percentile) of the genes with the highest combined centrality (betweeness centrality * outdegree) are shown. The six genes that appear in two lists (hsa-miR-181a, hsa-miR-423-3p, hsa-miR-1225-5p, hsa-miR-1268, SNORA40 and SNORD23) are in bold.

### Differential networks

Considering that despite the high similarity in network topology between the status-specific networks the key ncRNAs seem to be different in each of the conditions, we decided to perform node to node and edge to edge comparisons between the networks with the aim of identifying which part of each is network is shared by the others.

**Figure 4:**
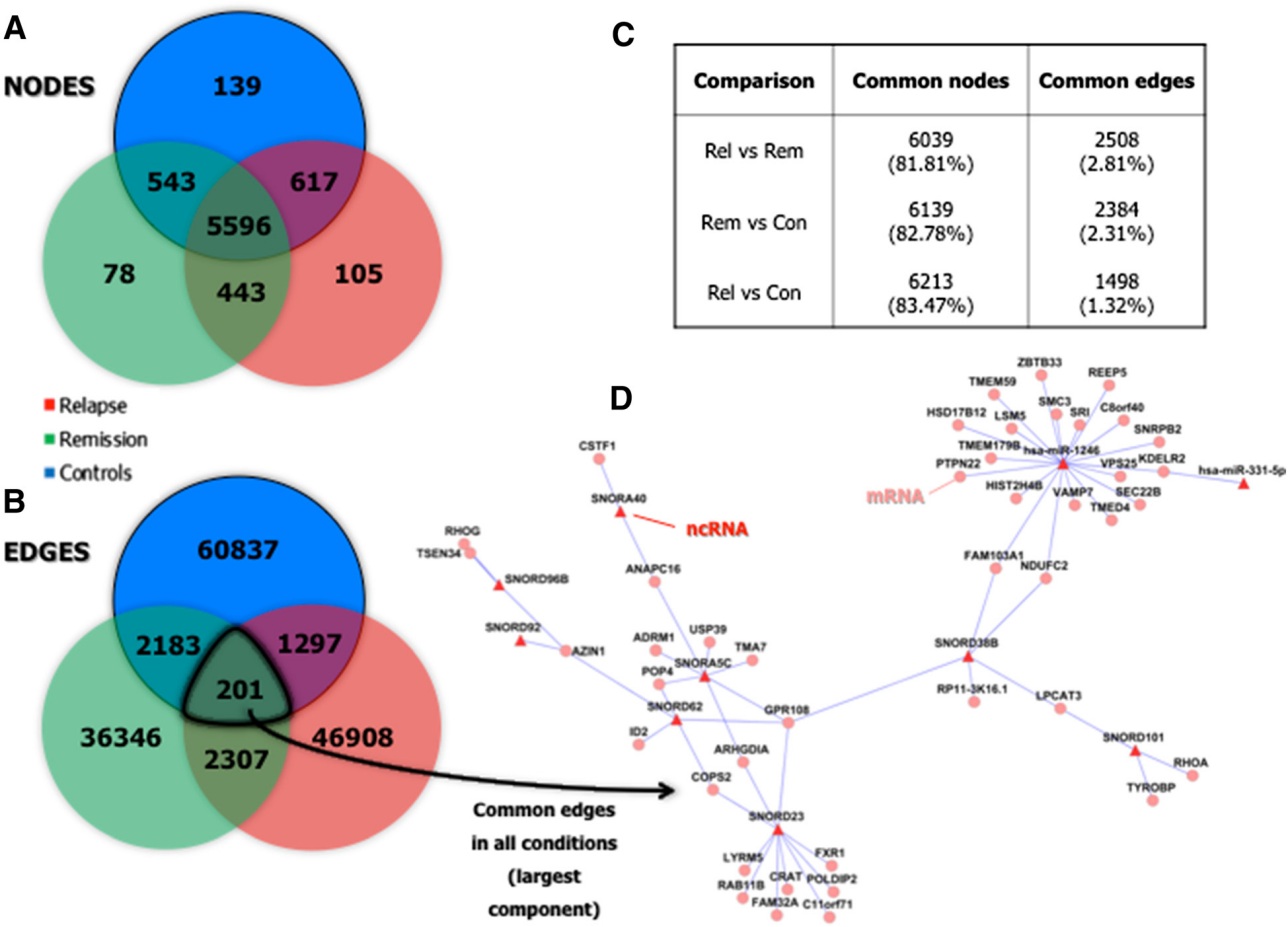
**Node to node and edge to edge comparisons of the ncRNA – mRNA networks obtained from the relapse, remission and control samples.** The venn diagrams show the number of nodes **(A)** and edges **(B)** shared by the three networks. The proportion of shared nodes/edges between status-specific networks is also shown **(C)**. Finally, the largest component (53 nodes/56 edges) of the network wired by the 201 edges that appear in all three networks is shown **(D)**.

The results of the node to node and edge to edge comparisons between the networks show that, whereas the three networks share a high proportion of their nodes (Figure 4A and C), they share very few edges (Figure 4B and C). This suggests that, even though the players of the coexpression network and the general topology are maintained between the different disease statuses, the relations between genes change in most of the cases. Based on that, it seems that a massive rewiring of the ncRNA – mRNA correlation network happens between conditions, which, although has little impact in topological features, provokes a big change in which ncRNAs play a central role in each of the networks (Table 1).

On the other hand, from the 150079 unique edges that appear in any of the three networks, only 201 (0.13%) appear in the three networks (Figure 4B). The largest component of the network wired by these 201 edges, is a 53 node/56 edge network (Figure 4D) that represents a status-independent core network that appears in peripheral blood leukocytes irrespective of disease status. As it could be expected, six of the ten ncRNAs that provide the backbone for this network have appeared as being central for network topology in previous lists: hsa-miR-331-5p, hsa-miR-1246, SNORA5C, SNORA40, SNORD23 and SNORD62 (Figure 2C and Table 1).

**Figure 5:**
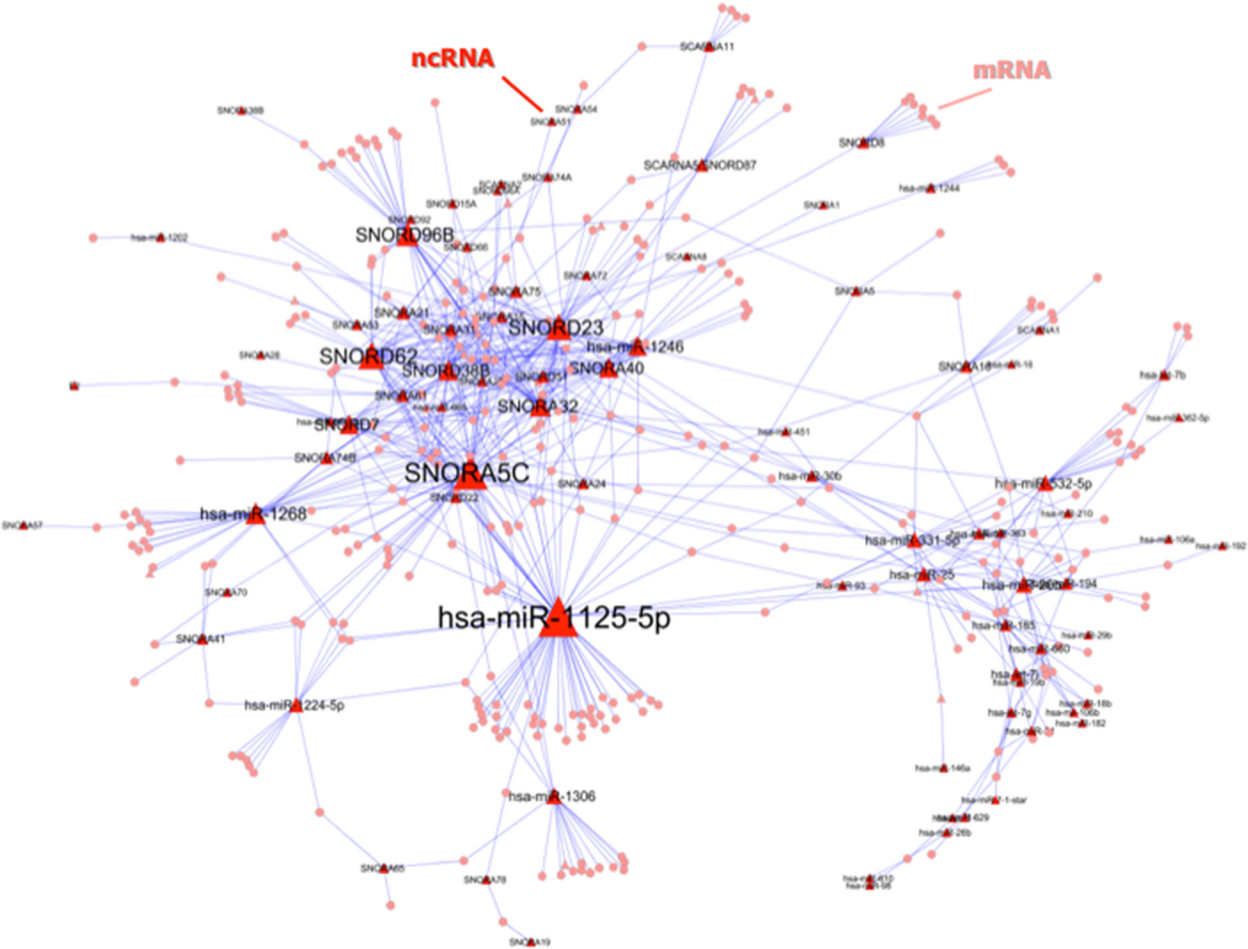
**Largest component (401 nodes/742 edges) of the network built from the 2307 edges shared by the relapse and remission networks but not the controls’ network.**

Finally, a network has been constructed with the 2307 edges shared by the relapse and remission networks, but not the controls’ network (Figure 4B), with the aim of identifying a disease-specific network that could guide the detection of ncRNAs with potential to be used as therapeutic targets. The largest component of the resulting network is composed by 401 nodes and 742 edges (Figure 5).

### Therapeutic target candidate selection

From the ncRNAs that appear in this last network, the ones that in a previous differential expression analysis (miRNEM study, results not published) were found to be as differentially expressed in relapse (vs. remission) or in remission (vs. controls), have been selected as potential candidates for therapeutic intervention (Table 2). The rationale behind this is that the ncRNA whose expression has been found to be significantly altered in MS (in relapse or remission, or both) and that is part of a disease-specific network must presumably have a relevant role in the physiopathology of the disease and, thus, it can be a good therapeutic target candidate.

**Table 2:** **List of potential therapeutic ncRNA targets selected from the disease-specific network and that, in a previous analysis (results not published), were identified as differentially expressed genes either in relapse (vs. remission), in remission (vs. controls) or in both conditions**

**ncRNA**	**DEG_REL**	**DEG_REM**	**Outdegree**
SNORD62	yes	no	39
SNORA40*	yes	yes	24
hsa-miR-1246	yes	yes	22
hsa-miR-20b	yes	no	17
hsa-miR-331-5p	yes	no	15
hsa-miR-1224-5p	yes	no	14
SNORA15	yes	no	7
hsa-miR-660	no	yes	7
SNORA24	yes	no	6
hsa-miR-21	no	yes	4
hsa-miR-26b	yes	yes	2
hsa-miR-18b	yes	yes	2
hsa-let-7f	no	yes	2
SNORA70	yes	no	2
hsa-miR-210	yes	yes	2
hsa-miR-1202	yes	no	2
hsa-miR-192	no	yes	1
hsa-miR-98	yes	no	1

**DEG_REL**: differentially expressed gene in relapse; **DEG_REM**: differentially expressed gene in remission. The ncRNAs that have previously appeared as key connectors in the global network and/or the controls’ network and/or are part of status-independent network are underlined. SNORA40 (marked*) is a good therapeutic target candidate that must be manipulated with care.

The selection has produced a list with 18 ncRNA candidates with potential to be used as therapeutic targets. However, it must be taken into account that two of them, SNORD62 and hsa-miR-1246, have been revealed to have a key role in the global network and are part of the core status-independent network, suggesting that their manipulation would have a large impact in the transcriptome of in peripheral blood leukocytes and, thus, a therapeutic approach based on their manipulation could produce many effects beyond the desired ones. Besides, SNORA40 has also appeared both in the controls’ network as an important connector and in the core condition-independent network. However, it does not hold a prime position among the top ncRNAs of the controls’ network (18^th^ highest combined centrality) and its connectivity is very low in the core network (outdegree = 2). Thus, although it must be manipulated with care, we still consider SNORA40 one of the best candidates for a hypothetical therapeutic intervention.

## Discussion

With the aim of studying the coexpression relations between selected ncRNAs (miRNAs, snoRNAs and sdRNAs) and mRNAs and the effect that the diseased-state of multiple sclerosis exerts on those relations, ncRNA – mRNA negative correlation networks have been built from both whole genome mRNA and ncRNA expression data obtained from MS patients in relapse and remission and healthy controls. Condition-specific networks (relapse, remission and healthy controls’ networks) have been built and compared in order to identify the parts of the system most perturbed by the disease. Finally, the most central ncRNAs of the perturbed network have been identified with the aim of obtaining a list of candidate therapeutic targets.

The study has been performed in peripheral blood leukocytes isolated from patients with a disease where the most affected system is the central nervous system, what might look contradictory. It needs to be kept in mind, however, that a fraction of the immune cells that execute the aberrant immunological attack against the myelin migrate from peripheral blood to the CNS through a disrupted blood–brain barrier [27]. Besides, most of the disease-modifying treatments used today against RRMS are based on immunomodulatory drugs that target the immune cells of peripheral blood and that, although not capable of completely stopping the progression of the disease, produce a big decrease in the relapse rate, pointing at an important pathophysiological function in MS for those cells [28]. Evidence suggests that several types of leukocytes (mainly B cells, Th17 cells, Th1 cells and macrophages) participate in the immunological attack that produces the lesions [29].

### Scale-free topology

In accordance to previous large-scale coexpression studies, the node-degree distribution of the ncRNA – mRNA networks we have built for the present study follows a negative power law in all cases, suggesting a scale-free topology. After the appearance of the first large-scale coexpression networks in the early 2000s, their scale-free topology was described soon. Apart from isolated exceptions where a negative exponential-law was found to be a better fit [30], the node-degree distributions of most large-scale coexpression networks built for organisms as different as *Saccharomyces cerevisiae* [31,32], mice [33] and human [33-35] and diseased states like gastric cancer [36], have been found to follow a negative power law. Besides, although the argument of the power-law has been shown to lie between −3 and −2 for metabolic and protein interaction networks [36], it has been demonstrated that it lies between −2 and −1 for coexpression networks [31,34,36], which coincides with the values we have obtained (from −1.484 to −1.260).

In contrast, the coexpression network we created for a differentially expressed gene set in a previous study did not show a scale-free topology [20]. It has to be kept in mind, however, that the scale-free topology property of coexpression networks appears when we analyze the whole system or a substantial part of it and that we may not observe this property when constructing small networks using, for example, only the genes found to be deregulated in a differential expression analysis. The scale-free property may be lost if the part of the system we are studying is small enough. Besides, the genes identified as upregulated/downregulated in a differential expression analysis tend to form tightly coregulated groups and give rise to extremely clustered and non-scale-free coexpression networks.

### Massive rewiring between conditions

As we have commented previously, it has been suggested that, to take profit of all the power of network analysis in biological studies, we need to take into account that biological networks are not constant across organisms, tissues, physiological states etc., but suffer substantial changes between different conditions [37]. A recent study has shown that the addition of a DNA damaging agent (methyl-methane-sulfonate (MMS)) produces a massive rewiring of the genetic functional interaction network in yeast [38]. The authors created genetic networks based on functional interactions between the 418 genes of yeast for both the untreated and the MMS-treated conditions and observed that from the 3126 unique interactions present in any of the two networks, 2050 (65.56%) were condition-specific, demonstrating that the sole addition of a DNA-damaging chemical agent produces a massive rewiring of the network.

Besides, one of the major goals of systems biomedicine is the identification of specific gene-regulatory networks that are dysfunctional in a given diseased state. Although we are not capable of performing proper differential gene regulatory network analyses yet, some steps in this direction have been taken such as, for example, the identification of differential coexpression networks [39]. As for the genetic functional interaction network created in yeast, some evidence suggests that coexpression networks also undergo massive rewiring between different conditions. For example, it has been demonstrated that when comparing large-scale coexpression networks created for mice and humans, although the architecture of the networks is very similar, a high level of divergence exits at the local level, as less than 10% of all unique connections are shared by the networks from both species [35].

We observe a similar phenomenon in the condition-specific (relapse-, remission- and control-specific) ncRNA – mRNA coexpression networks we have created for the differential network analysis. Although the topological features of the three networks are practically identical, like in the mouse/human example, a very low fraction of edges or connections appear in more than one network. From the 150079 unique connections present in any of the three networks, only 201 (0.13%) are shared by the three graphs and the fraction of edges shared by any pair of networks lies between 1.32% and 2.81%. These results indicate that the two diseased-states of multiple sclerosis, the relapse and the remission, have a strong effect on the coexpression relations between the selected ncRNAs and mRNAs and produce a massive rewiring of the ncRNA – mRNA network. It seems that the topological features of coexpression networks are held to constrains that are constant across species, organisms, tissues and physiological-states but, inside the limits of network-topology, transcriptomic networks have great flexibility to respond by changing interactions between genes.

### snoRNAs in gene silencing

After the first characterizations of rRNA-bound small nucleolar RNAs in the mid-70s [40], the two main snoRNA types that have been characterized, the H/ACA box and C/D box snoRNAs, have been widely considered to be involved in an only function, the guidance of post-transcriptional modifications of rRNA and other target-RNAs performed by the snoRNP complexes. However, our results suggest that many snoRNAs may have a relevant role in post-transcriptional regulation of gene expression, as revealed by the fact that many of them present a high number of links to mRNAs and hold central positions in the several ncRNA – mRNA coexpression networks we created. This apparent widespread function of snoRNAs in gene silencing cannot be explained by their well-established involvement in the splicing machinery and in rRNA modification.

Nevertheless, in an immunoprecipitation followed by deep-sequencing experiment on RNAs associated to Ago1 and Ago2 (argonaute proteins of the gene-silencing effector complexes), Ender and collegues found evidence of small non-coding RNAs derived from SNORA45 (sdRNAs), whose processing happens in a drosha-independent, dicer-dependent manner [11]. They predicted CDC2L6 as a potential target for the sdRNA and found evidence for snoRNA-derived RNAs with miRNA-like processing signatures and a potential role in post-transcriptional gene silencing for 7 additional snoRNAs, [11].

That discovery opened the possibility of snoRNAs having, apart from their involvement in RNA modification guidance, an additional function in gene silencing through their role as precursors for sdRNAs, also called sno-miRNAs [41]. Since 2008, several works have explored the potential role of snoRNAs in miRNA-like small RNA production and the evolutionary/molecular relationships between snoRNAs and miRNAs. More work has been done in the direction set by Ender et al., and additional snoRNA-derived RNAs have been identified by deep sequencing and their target-gene silencing functions have been experimentally validated in many cases [42]. For example, Scott et al., in a completely computational approach, tested whether the precursors of known miRNAs show any SNORA-like (H/ACA box snoRNA) features and found evidence of at least 20 known miRNA precursors having sequence/conformational similarities with SNORAs. They also observed that the genomic regions surrounding these SNORA-like miRNA precursors resemble the regions around snoRNA retrotransposons, suggesting a close evolutionary relationship between SNORAs and miRNAs [43]. Similar findings have been made when applying this approach to C/D box snoRNAs [44]. It has recently been proposed a model where the RNA species we include in the snoRNA and miRNA families would form a spectrum from classical snoRNAs to prototypical miRNAs: classical snoRNAs ↔ snoRNAs with miRNA-like features ↔ dual function sno-miRNAs ↔ miRNAs with snoRNA-like features ↔ prototypical miRNAs [41].

It is clear, thus, that the precursors of many known miRNAs have snoRNA-like features and that many snoRNAs present miRNA precursor features and are involved in miRNA or miRNA-like RNA production in a Drosha-independent Dicer-dependent manner. This indicates that snoRNAs probably have a relevant role in the post-transcriptional downregulation of gene expression through the involvement of their product sdRNAs (snoRNA-derived RNAs or sno-miRNAs) in the gene silencing effector complexes.

However, our results suggest that this sdRNA-precursor and gene silencing role of snoRNAs is not a rare event but a widespread phenomenon affecting a big fraction of snoRNAs. From the 169 snoRNAs (87 H/ACA box and 82 C/D box snoRNAs) left for subsequent analysis after the initial filtering of the miRNA array data, 118 (69.82%) appear in the global network as negatively correlated with mRNAs. This result concurs with previous estimations of around 60% of human snoRNAs showing evidence of being precursors of highly conserved sdRNAs with a potential miRNA-like function [45].

### A consistent central role for hsa-miR-1246

Our results point at hsa-miR-1246 as having a consistent central position in the ncRNA – mRNA coexpression network of peripheral blood leukocytes, as it appears to be one of the key connectors in the global network, the controls’ network and the core status-independent network. These results suggest that miR-1246 may hold a central position in the gene regulatory network of leukocytes.

However, as it happens with many miRNAs, the role of miR-1246 has mostly been studied in relation to diseases, especially cancer. It has been shown that miR-1246 is one of the mediators of the pro-apoptotic activity of p53 and its analogs p63 and p73. The transcription of miR-1246 is activated by these genes and miR-1246, in turn, downregulates DYRK1A, a Down syndrome-associated protein kinase, establishing a link between cancer and Down syndrome [46,47]. This miRNA has also been found to be upregulated in hepatoma cells upon infection with the hepatitis C virus [48]. Apart from cancer, miR-1246 has been shown to be involved in the regulation of chloride transport in epithelial cells by targeting CFTR and SLC12A2 [49] and in the regulatory phenotype of T cells [50].

Interestingly, miR-1246 has been identified in extracellular vesicles (microvesicles, microparticles and exosomes) derived from a wide range of cell types and cell lines. This miRNA has been found in microparticles derived from platelets [51], in exosomes released by malignant epithelial cells [52] and in microparticles and microvescicles derived from a variety of cell lines like THP-1 (a monocytic cell-line derived from an acute monocytic leukemia patient) and HUVEC (a cell line derived from the endothelium of the veins of the umbilical cord) [51].

### Top therapeutic target candidate selection

The selection of potential therapeutic target ncRNAs has been done based on the premise that if a ncRNA appears in the multiple sclerosis-specific coexpression network and, in addition, has been shown to be differentially expressed in any or both of the phases of the disease (relapse and remission) (unpublished results from the miRNEM study) it must have a relevant role in the physiopathology of the disease.

Among the selected ncRNAs, the best candidates for a therapeutic intervention are those with a high outdegree in the disease-specific network and, thus, SNORA40, miR-20b, miR-331-5p and miR-1224-5p appear to be the most prominent candidates. Since the revival of network analysis techniques for the modeling of biological systems, several groups have found evidence that the connectivity of a gene in a biological network is a good prediction of the essentiality of the gene for the organism and the probability of its removal having lethal consequences. In 2001, Jeong et al., demonstrated that the phenotypic consequence of a single gene deletion in *Sacchromyces cerevisiae* is affected to a large extent by the topological position of its protein product in the complex hierarchical web of molecule interactions [53]. They concluded that “the most connected proteins in the cell are the most important for its function”. Since then, this correlation between the centrality of a gene in a gene-network and its essentiality has been observed in several types of biological networks and organisms. It has been shown that the proteins with a central position in the protein interaction networks of yeast (*S. cerevisiae*), worm (*C. elegans*) and fly (*D. melanogaster*) evolve slowly and are more likely to be essential for survival [54] and similar observations have been made on a *C. elegans* genetic network constructed by integrating coexpression at the RNA level, interactions at the protein level and co-citations between genes in the literature [55]. In front of all this evidence, thus, it seems evident that the connectivity of a gene in a genetic network (whether it is a protein interaction network, a gene functional network or a coexpression network) is strongly correlated with its essentiality and and this is why we use centrality measures to interpret the importance that miRNAs and snoRNAs may have both for normal physiology (in the global and controls’ networks) and for multiple sclerosis pathophysiology (in the relapse and remission networks and, specially, in the disease-specific network).

Besides, several works have demonstrated that the removal of miRNA genes has, in some cases, a strong effect on the phenotype of mice (reviewed in [56]). Although the creation of knockout mice for some miRNAs does not produce any apparent changes on phenotype (as is the case for miR-106a-363, miR-208b, miR-499 and miR-140, among others), the removal of other miRNAs has been shown to have a strong effect on the phenotype of the knockout mouse in question. For example, the miR-1-2 knockout mouse presents a mortality of 50% with cardiac defects; the removal of miR-155 produces an impairment of T-cell and B-cell dependent immunity; and the knockout mouse for the islet-expressed miR-375 suffers hyperglycemia.

Taking into account that, on one hand, the centrality of genes in molecular networks is strongly correlated to their essentiality and, on the other hand, that the removal of non-coding genes such as miRNAs can exert a strong effect on the phenotype of a higher organism such as mice, we think that SNORA40, miR-20b, miR-331-5p and miR-1124-5p are the best candidates to be considered as therapeutic target ncRNAs in MS. Following the same logic, SNORD62 and miR-1246 should not be manipulated in a hypothetical therapeutic strategy as they seem to have a consistent central role in conditions of physiological normality, as revealed by their central positions both in the global and the controls’ ncRNA – mRNA coexpression networks.

### SNORA40

Based on its central role in the disease-specific network and the differential expression detected for it in the miRNEM study, the ncRNA that, above all the rest, seems to be the most prominent candidate for a hypothetical therapeutic intervention is SNORA40. This is the ncRNA that, apart from SNORD62 (which also has central roles in the global and controls’ networks), shows the highest outdegree in the disease-specific network. It is true that it appears in the list of top connectors for the controls’ network and is also part of the core status-independent network. Nevertheless, it does not hold a prime position among the top connectors of the controls’ network and its connectivity is very low in the core network. Besides, it has been identified as deregulated both in relapse and remission, showing a fold-change of 9.21 in relapses of female patients (unpublished data from miRNEM). Thus, we consider that, although it must be manipulated with care, SNORA40 is a good candidate target for a hypothetical therapeutic intervention.

This H/ACA box small-nucleolar RNA has been shown to be involved in cancer and senescence. SNORA40 has been seen to be overexpressed in plasma cells (antibody-secreting B cells) from patients with multiple myeloma of the TC1 subgroup when compared to those from healthy controls [57]. In another work aimed at the characterization of the molecular mechanisms of senescence, a RNA-seq experiment was performed on RNA isolated from the cartilage of young (<4 years) and elder (<15 years) horses and SNORA40 was found to be overexpressed in the cartilage of the elder donors [58].

Even more interestingly, alterations in the expression of this snoRNA have been observed in relation to normal and aberrant immunological responses. It has recently been studied the alteration of the transcriptional profile of NK92 cells (a cell line with characteristics of natural killer cells) when co-cultured with red blood cells infected with the malaria agent *Plasmodium falciparium* in order to shed light on the molecular mechanisms implicated in the response of natural killer cells to the infection by this pathogen. The authors found that SNORA40 was significantly overexpressed in the NK92 cells responding to the infected red blood cells, suggesting that it may have a relevant role in the natural killer cell response to *P. falciparium* infection [59]. Besides, SNORA40, along with other snoRNAs and olfactory receptor genes, was found to be part of a coexpression module composed of genes upregulated in exacerbations of children with asthma [60]. The results of this last study overlap surprisingly well with the results we obtained in the GEXEM study [20], as we identified two coexpression modules composed mainly of small non-coding RNAs (in females) and olfactory receptors (in males) among the genes upregulated in relapses. All this evidence suggests that SNORA40 may have a consistent and relevant role in aberrant responses of the immune system such as allergic (asthma) and autoimmune (multiple sclerosis) responses.

## Conclusions

Our results indicate that the two diseased states of multiple sclerosis, relapse and remission, have a strong impact on the organization of the ncRNA – mRNA coexpression network of the peripheral blood leukocytes, as we have observed a massive rewiring of the network between conditions. Besides, the consitent and prominent presence of snoRNAs among the top regulators of the several networks built in the present work points at the previously described function at targeted downregulation of gene expression of these ncRNAs as being a widespread phenomenon. Finally, the combination of differential expression and differential coexpression analyses has allowed the identification of miR-20b, miR-331-5p, miR-1246 and SNORA40 as potential theraputic targets in multiple sclerosis, SNORA40 being the promising candidate.

## Additional file

Additional file 1: Figure S1. Global sncRNA – mRNA positive correlation network. This network presents an only fully-connected component composed by 6069 nodes and 56461 positive sncRNA – mRNA positive correlations (Pearson’s R > 0.42) **(A)**. The node degree distribution is represented in a logarithmic scale in both axes and fits a negative power law **(B)**. The 22 sncRNAs with a combined centrality above percentile 0.975 (Combined centrality > 1.6) are listed **(C)**. **Table S1.** Sex, age and MS-specific treatment of the subjects included in the study.
