# Supplementary material for: Identification of ncRNAs as potential therapeutic targets in multiple sclerosis through differential ncRNA – mRNA network analysis
Source: BMC Genomics. 2015 Mar 28;16(1):250. doi: 10.1186/s12864-015-1396-5 (PMC4391585; doi:10.1186/s12864-015-1396-5)
Supplement: Additional file 1: Figure S1. — Global sncRNA – mRNA positive correlation network. This network presents an only fully-connected component composed by 6069 nodes and 56461 positive sncRNA – mRNA positive correlations (Pearson’s R > 0.42) (A). The node degree distribution is represented in a logarithmic scale in both axes and fits a negative power law (B). The 22 sncRNAs with a combined centrality above percentile 0.975 (Combined centrality > 1.6) are listed (C). Table S1. Sex, age and MS-specific treatment of the subjects included in the study. [file 12864_2015_1396_MOESM1_ESM.docx]

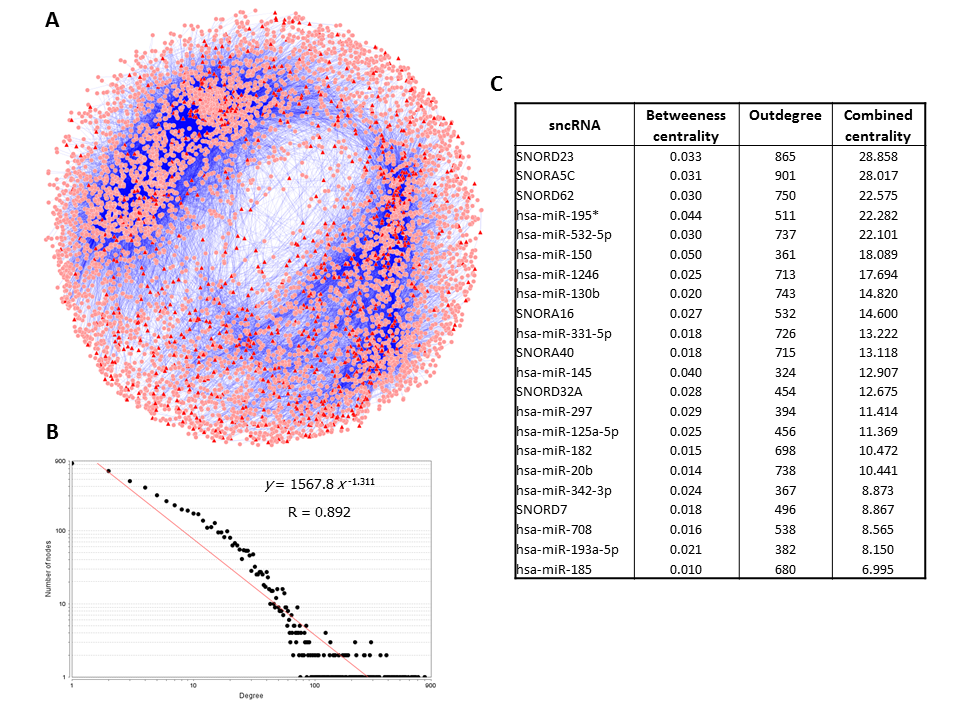


**Figure 1**: global sncRNA – mRNA positive correlation network. This network presents an only fully-connected component composed by 6069 nodes and 56461 positive sncRNA – mRNA positive correlations (Pearson’s R > 0.42) (**A**). The node degree distribution is represented in a logarithmic scale in both axes and fits a negative power law (**B**). The 22 sncRNAs with a combined centrality above percentile 0.975 (Combined centrality > 1.6) are listed (**C**).

**Table 1**: Sex, age and MS-specific treatment of the subjects included in the study.

|  | **Subject** | **Sample** | **Condition** | **Sex** | **Age range (yrs)** | **MS treatment** |
| --- | --- | --- | --- | --- | --- | --- |
| **MS PATIENTS IN RELAPSE AND REMISSION** | 00-024 | 00-024BM | Relapse | Female | 45-50 | - |
|  |  | 00-024NBM | Remission |  | 45-50 | - |
|  | 00-096 | 00-096BV | Relapse | Male | 55-60 | - |
|  |  | 00-096NBV | Remission |  | 55-60 | - |
|  | 00-129 | 00-129BM | Relapse | Female | 25-30 | Tysabri |
|  |  | 00-129NBM | Remission |  | 25-30 | Tysabri |
|  | 04-074 | 04-074BV | Relapse | Male | 40-45 | Copaxone |
|  |  | 04-074NBV | Remission |  | 40-45 | Copaxone |
|  | 05-004 | 05-004BM | Relapse | Female | 30-35 | - |
|  |  | 05-004NBM | Remission |  | 30-35 | Betaferon |
|  | 05-007 | 05-007BM | Relapse | Female | 30-35 | Copaxone |
|  |  | 05-007NBM | Remission |  | 30-35 | Copaxone |
|  | 06-185 | 06-185BM | Relapse | Female | 35-40 | - |
|  |  | 06-185NBM | Remission |  | 35-40 | Rebif44 |
|  | 07-254 | 07-254BM | Relapse | Female | 45-50 | Tysabri |
|  |  | 07-254NBM | Remission |  | 40-45 | Tysabri |
|  | 07-309 | 07-309BV | Relapse | Male | 25-30 | - |
|  |  | 07-309NBV | Remission |  | 25-30 | Rebif 44 |
|  | 08-111 | 08-111BV | Relapse | Male | 45-50 | Avonex |
|  |  | 08-111NBV | Remission |  | 45-50 | Avonex |
|  | 08-377 | 08-377BM | Relapse | Female | 50-55 | Avonex |
|  |  | 08-377NBM | Remission |  | 50-55 | - |
|  | 09-152 | 09-152BM | Relapse | Female | 45-50 | Betaferon |
|  |  | 09-152NBM | Remission |  | 45-50 | Imurel |
|  | 09-394 | 09-394BV | Relapse | Male | 40-45 | - |
|  |  | 09-394NBV | Remission |  | 40-45 | - |
|  | 09-475 | 09-475BV | Relapse | Male | 40-45 | Copaxone |
|  |  | 09-475NBV | Remission |  | 40-45 | Copaxone |
|  | 10-333 | 10-333BM | Relapse | Female | 45-50 | Tysabri |
|  |  | 10-333NBM | Remission |  | 45-50 | Rebif44 |
|  | 10-336 | 10-336BV | Relapse | Male | 20-25 | - |
|  |  | 10-336NBV | Remission |  | 20-25 | Copaxone |
|  | 10-360 | 10-360BV | Relapse | Male | 30-35 | Copaxone |
|  |  | 10-360NBV | Remission |  | 35-40 | Copaxone |
|  | 10-361 | 10-361BM | Relapse | Female | 35-40 | Betaferon |
|  |  | 10-361NBM | Remission |  | 35-40 | Betaferon |
|  | 10-700 | 10-700BM | Relapse | Female | 25-30 | - |
|  |  | 10-700NBM | Remission |  | 30-35 | Copaxone |
|  | 11-338 | 11-338BV | Relapse | Male | 40-45 | - |
|  |  | 11-338NBV | Remission |  | 40-45 | - |
|  | 11-353 | 11-353BM | Relapse | Female | 25-30 | - |
|  |  | 11-353NBM | Remission |  | 25-30 | Rebif44 |
|  | 99-276 | 99-276BV | Relapse | Male | 35-40 | Betaferon |
|  |  | 99-276NBV | Remission |  | 35-40 | Betaferon |
| **HEALTHY CONTROLS** | 08-584 | 08-584CV | Control | Male | 55-60 |  |
|  | 08-588 | 08-588CM | Control | Female | 30-35 |  |
|  | 08-647 | 08-647CV | Control | Male | 30-35 |  |
|  | 08-650 | 08-650CM | Control | Female | 20-25 |  |
|  | 08-659 | 08-659CM | Control | Female | 35-40 |  |
|  | 08-759 | 08-759CV | Control | Male | 40-45 |  |
|  | 09-1203 | 09-1203CM | Control | Female | 25-30 |  |
|  | 09-221 | 09-221CV | Control | Male | 25-30 |  |
|  | 09-708 | 09-708CM | Control | Female | 40-45 |  |
|  | 09-880 | 09-880CV | Control | Male | 20-25 |  |
|  | 09-883 | 09-883CV | Control | Male | 35-40 |  |
|  | 09-900 | 09-900CV | Control | Male | 45-50 |  |
|  | 09-901 | 09-901CV | Control | Male | 50-55 |  |
|  | 09-902 | 09-902CV | Control | Male | 45-50 |  |
|  | 10-321 | 10-321CM | Control | Female | 35-40 |  |
|  | 11-502 | 11-502CM | Control | Female | 45-50 |  |
|  | 11-504 | 11-504CM | Control | Female | 30-35 |  |
|  | D1 | D1CV | Control | Male | 40-45 |  |
|  | D17 | D17CM | Control | Female | 30-35 |  |
|  | D18 | D18CM | Control | Female | 35-40 |  |
|  | D6 | D6CM | Control | Female | 40-45 |  |
